# Two-photon FLIM of NAD(P)H and FAD in mesenchymal stem cells undergoing either osteogenic or chondrogenic differentiation

**DOI:** 10.1186/s13287-017-0484-7

**Published:** 2017-01-28

**Authors:** Aleksandra V. Meleshina, Varvara V. Dudenkova, Alena S. Bystrova, Daria S. Kuznetsova, Marina V. Shirmanova, Elena V. Zagaynova

**Affiliations:** 1 Institute of Biomedical Technologies, Nizhny Novgorod State Medical Academy, Minin and Pozharsky Square, 10/1, Nizhny Novgorod, 603005 Russia; 2 Institute of Biology and Biomedicine, Nizhny Novgorod State University, Gagarin Avenue, 23, Nizhny Novgorod, 603950 Russia; 3 Department of Radiophysics, Nizhny Novgorod State University, Gagarin Avenue, 23, Nizhny Novgorod, 603950 Russia

**Keywords:** Mesenchymal stem cells, NAD(P)H, FAD, Osteogenic and chondrogenic differentiation, Metabolic shift, Two-photon fluorescence microscopy, FLIM

## Abstract

**Background:**

Metabolic plasticity and the versatility of different lineages of stem cells as they satisfy their energy demands are not completely understood. In this study we investigated the metabolic changes in mesenchymal stem cells (MSCs) undergoing differentiation in two directions, osteogenic and chondrogenic, using two-photon fluorescence microscopy combined with FLIM.

**Methods:**

Differentiation was induced by incubating the human bone marrow MSCs in osteogenic or chondrogenic mediums. Cellular metabolism was examined on the basis of the fluorescence of the metabolic cofactors NAD(P)H and FAD. The optical redox ratio (FAD/NAD(P)H) and the fluorescence lifetimes of NAD(P)H and FAD were traced using two-photon fluorescence microscopy combined with FLIM. The cells were imaged before the induction of differentiation (day 0) and on days 7, 14, and 21 of osteogenic and chondrogenic differentiation.

**Results:**

Based on the data for the FAD/NAD(P)H redox ratio and on the fluorescence lifetimes of protein-bound NAD(P)H, we registered a metabolic shift toward a more glycolytic status in the process of MSC differentiation. The difference was that, in osteogenic differentiation, an increase in oxidative phosphorylation preceded the shift to the glycolytic status in the process of such MSC differentiation. The fluorescence lifetime characteristics of FAD indicated the stimulation of an unknown metabolic pathway, where protein-bound FAD participates.

**Conclusions:**

In this study, probing of the metabolic status of MSCs during osteogenic and chondrogenic differentiation was implemented for the first time with the use of optical metabolic imaging of the two cofactors - NAD(P)H and FAD. Our data suggest that biosynthetic processes, associated, presumably, with the synthesis of collagen, drive energy metabolism in differentiating cells, and promote a metabolic shift from a more oxidative to a more glycolytic state.

## Background

Fluorescence lifetime imaging microscopy (FLIM) is widely used in biomedical science as it offers non-invasive, real-time measurements, optical sectioning capability with high photon efficiency, high lifetime accuracy, and simultaneous recording at several wavelength intervals. FLIM images represent a spatial distribution through the fluorescence decay profiles in every pixel. Several fluorescence detection methods are available for lifetime measurements, of which, time-correlated single photon counting (TCSPC) enables simple data collection and enhanced quantitative photon counting. The frequency domain method involves sinusoidal modulation of the incident light at high frequencies. In this method, the emission occurs at the same frequency as the incident light, but accompanied by a phase delay and a change in the amplitude relative to the excitation light (demodulation) [1].

Among FLIM applications, the imaging of endogenous fluorophores, such as nicotinamide adenine dinucleotide (NAD(P)H) and flavin adenine dinucleotide (FAD) has undeniable value for metabolic studies because it does not require any specific labeling [2]. Importantly, these coenzymes are naturally fluorescent and, therefore, genuine, non-invasive imaging of metabolic activities can be carried out in living cells and tissues [3].

As the fluorescence lifetime of a fluorophore depends on its molecular environment but not on its concentration, FLIM of NAD(P)H and FAD allows us to distinguish between the free and protein-bound forms of the cofactors [4, 5]. The short and long lifetime components in NAD(P)H fluorescence decay are associated with free and protein-bound states, respectively. FAD has both short and long lifetime components, depending, respectively, on whether it is protein-bound or free [6, 7].

NAD(P)H and FAD are major indicators of the emphasis of cellular metabolism. NADH and its oxidized form (NAD+) are involved in mitochondrial function, energy metabolism, calcium homeostasis, gene expression, oxidative stress, aging, and apoptosis. NAD+(NADH) drives adenosine triphosphate (ATP) production in the cytosol by glycolysis, and in the mitochondria by oxidative phosphorylation. The reduced phosphorylated form, NADPH, is involved in the reductive biosynthesis of fatty acids and steroids, antioxidation and oxidative stress, while the oxidized form, NADP+, participates in calcium homeostasis [8].

Although FAD-containing proteins participate in a variety of metabolic pathways including electron transport, DNA repair, nucleotide biosynthesis, the beta-oxidation of fatty acids, and amino acid catabolism, as well as the synthesis of other cofactors such as coenzyme A (CoA), coenzyme Q (CoQ) and heme groups, FAD is largely associated with mitochondria and the oxidative phosphorylation pathway [3, 9]. Succinate dehydrogenase (complex II in the electron transport chain) requires covalently bound FAD to catalyze the oxidation of succinate to fumarate by coupling it with the reduction of ubiquinone to ubiquinol. The high-energy electrons from this oxidation are stored momentarily by reducing FAD to FADH_2_. The FADH_2_ then reverts to FAD, sending its two electrons through the electron transport chain. Some data suggest that the majority of flavin fluorescence is produced by lipoamide dehydrogenase (LipDH)-containing enzyme complexes, such as the pyruvate dehydrogenase complex (PDHC) and the alpha-ketoglutarate dehydrogenase complex.

NAD(P)H and FAD are critical for a broad array of oxidation–reduction (redox) reactions in living cells. The ratio of the intensities of their fluorescence, referred to as their “redox ratio”, is also a useful tool for real-time monitoring of the metabolic state of a cell [10]. Although this ratio is not a direct measure of the concentrations of these fluorophores, the fluorescence intensity is a relative measure of their concentrations [3].

In stem cell biology the role of cellular metabolism and macromolecular synthesis during differentiation has not been fully studied. Many types of stem cell rely on glycolysis for energy when undifferentiated, and then activate the mitochondrial process of oxidative phosphorylation (OxPhos) during differentiation [11]. The switch of energy source from glycolysis to aerobic metabolism is a hallmark of such differentiated cells [12]. However, several studies demonstrate that not all differentiations of stem cells are accompanied by an obvious metabolic shift [13, 14]. So the metabolic plasticity and the versatility of different lineages of stem cells as they satisfy their energy demands require further clarifications.

The current work was aimed at monitoring the metabolic changes in mesenchymal stem cells (MSCs) undergoing differentiation in two directions, osteogenic and chondrogenic. Cellular metabolism was examined on the basis of the fluorescence of the metabolic cofactors NAD(P)H and FAD. The optical redox ratio (FAD/NAD(P)H) and the fluorescence lifetimes of NAD(P)H and FAD were traced using two-photon fluorescence microscopy combined with FLIM.

## Methods

### Stem cell culture, osteogenic and chondrogenic differentiation

Human bone marrow MSCs were isolated from the bone marrow of normal donors, with their informed consent according to the institutional guidelines under the approved protocol. The MSCs were isolated as described previously [15]. MSCs were immunophenotypically characterized by flow cytometry on the Cell Lab Quanta SC (Beckman Coulter, Brea, CA, USA). The common markers to human MSCs (CD34, CD45, HLA-DR, CD105, CD44, CD54, CD73, CD90) were studied [15].

The MSCs were cultured in MesenCult™ MSC Basal Medium (Human) (Stemcell Technologies, Vancouver, BC, Canada) supplemented with 10% fetal bovine serum (FBS) (GE Healthcare Hyclone, Logan, UT, USA), 0.58 mg/ml L-glutamine (PanEco, Moscow, Russia) and 40 U/ml gentamicin. The cell culture was maintained at 37 °C in a 5% CO_2_, humidified atmosphere.

Differentiation was induced by incubating the MSCs in MesenCult™ Osteogenic Stimulatory Kit (Human) (Stemcell Technologies) or Stem MACS™ Chondro Diff Media (MASC, Miltenyi Biotec GmbH, Bergisch Gladbach, Germany). The medium was replaced every 3–4 days over the experimental period of up to 4 weeks.

Morphological changes were assessed by counting the number of cells with spindle-shaped and polygonal morphology at each differentiation stage. Osteogenic and chondrogenic differentiations were verified by staining calcifications of the extracellular matrix with Alizarin Red S (Sigma-Aldrich, St. Louis, MO, USA,) and acidic polysaccharides such as glycosaminoglycans in cartilage with Alcian blue (Sigma-Aldrich), respectively.

For microscopic imaging, 4*10^5^ cells were transferred into a sterile dish with a cover glass bottom (0.17-mm thick) and incubated for 1 day until they attached to the glass surface. Cell monolayers were used. The cells were imaged before the induction of differentiation (day 0) and on days 7, 14, and 21 of osteogenic and chondrogenic differentiation. Untreated cells served as controls and were imaged on the same days of culturing. The cells were washed twice using phosphate-buffered saline, and then placed in FluoroBrite™ DMEM (Gibco, Carlsbad, CA, USA) with 10% FBS and 0.58 mg/ml L-glutamine (PanEco) and 40 U/ml gentamicin.

### Multiphoton fluorescence microscopy and FLIM

The two-photon excited fluorescence intensity and FLIM images of NAD(P)H and FAD were obtained using an LSM 710 (Carl Zeiss, Oberkochen, Germany) inverted laser scanning confocal microscope equipped with a time-correlated single photon counting (TCSPC) system (Simple-Tau 152, Becker & Hickl GmbH, Berlin, Germany).

NAD(P)H and FAD fluorescence was excited with a Chameleon Vision II (Coherent, Santa Clara, CA, USA) Ti:Sa femtosecond laser, using an 80 MHz repetition rate and a pulse duration of 140 fs at wavelengths of 750 nm and 900 nm, respectively. Emission was detected in the ranges 455–500 nm for NAD(P)H, and 500–550 nm for FAD.

An average of 8000–10,000 photons was collected per decay curve. The absence of photobleaching was confirmed by the constant photon count rate during image acquisition.

The average power of the Ti:Sa laser was measured using a PM100A power meter (ThorLabs Inc., Newton, NJ, USA). An internal microscope reference, reporting the two-photon excitation efficiency, was used to control the laser power in all the experiments. The average power incident on the samples was approximately 6 mW.

A C-Apochromat 40x/1.2 water immersion objective was used for image acquisition.

The presence of collagen fibers was confirmed by Second Harmonic Generation (SHG) microscopy. SHG of collagen was excited at a wavelength of 750 nm and detected in the range 373–387 nm.

During the experiments the cells were maintained at 37 °C and 5% CO_2_ in an XL multi S Dark LS incubator (PeCon GmbH, Erbach, Germany).

### Optical redox ratio calculation

The optical redox ratio was defined as the fluorescence intensity of FAD divided by the fluorescence intensity of NAD(P)H. The redox ratio was calculated from corresponding two-photon fluorescence images of FAD and NAD(P)H after subtracting the background on a pixel by pixel basis using ImageJ 1.39p software (NIH, Bethesda, MD, USA).

### Fluorescence lifetime analysis

The fluorescence lifetimes and their contributions (free and protein-bound forms of NAD(P)H and FAD: t1-free NADH, t2-bound NAD(P)H, a1-free NADH, a2-bound NAD(P)H; t1-bound FAD, t2-free FAD, a1-bound FAD, a2-free FAD) for the regions of interest (ROIs) were calculated by finding the global minimum of the χ2 value. The mean values of χ2 and the fluorescence lifetimes in undifferentiated MSCs and in MSCs during differentiation were assessed in the cell cytoplasm. Bi-exponential fitting was used for the analysis of both cofactors. FLIM images were processed in SPCImage software (Becker & Hickl GmbH).

### Statistical analysis

For each time point of the differentiation activity, from 23 to 78 randomly selected cells and from 47 to 120 ROIs were inspected. A statistical analysis was performed using STATISTICA 64 software, version 10 (StatSoft Inc., Tulsa, OK, USA). Mean and standard deviation (SD) values were used to express the data. Differences in the mean values were tested for significance using the Student’s *t* test or the one-way ANOVA with Fisher’s post hoc test (*p* ≤ 0.05).

## Results

### Osteogenic and chondrogenic differentiation of MSCs

Immunophenotypic profile of human bone marrow MSCs was characterized by identification of common markers. Isolated MSCs were uniformly positive for CD105, CD90, CD73, CD44, CD54, and negative for CD34, CD45, HLA-DR (Table 1).

**Table 1:** Fluorescence-activated cell sorting analysis of MSCs

Cell marker	Marker availability, %
CD34 (sialomucin)	1.290 ± 0.001
CD44 (hyaluronic acid receptor)	90.050 ± 0.006
CD45 (leukocyte common antigen)	0.460 ± 0.001
CD73 (5′-terminal nucleotidase)	93.030 ± 0.040
CD90 (Thy-1)	98.880 ± 0.001
CD54 (intercellular adhesion molecule 1)	47.830 ± 0.014
CD105 (endoglin)	97.130 ± 0.002
HLA-DR (major histocompatibility complex II)	1.600 ± 0.001

The data are expressed as mean ± SD. n = 3

*MSCs* mesenchymal stem cells

**Fig. 1:**
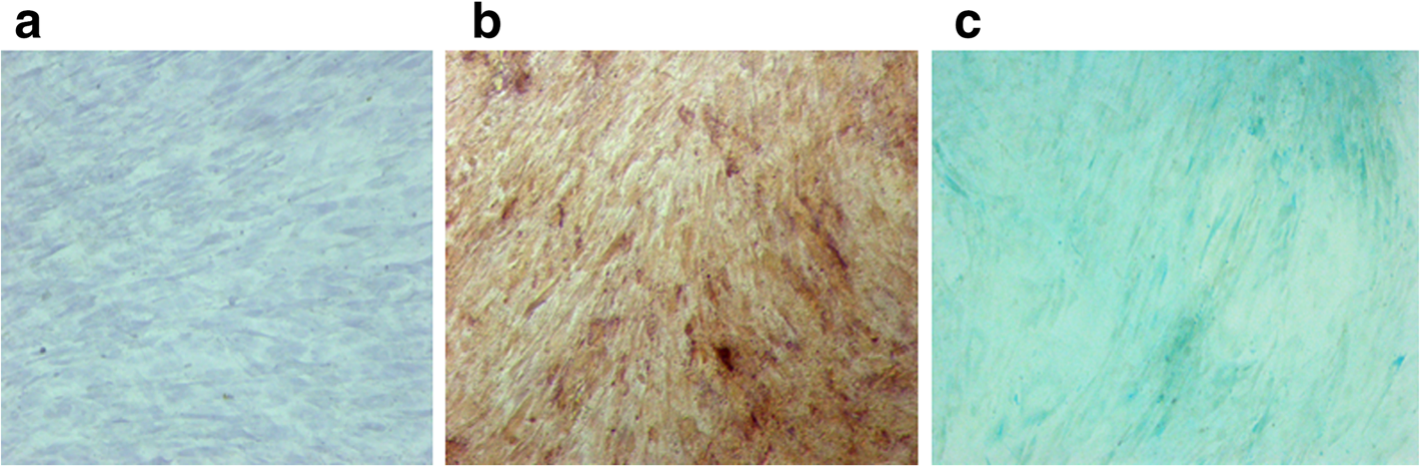
Microscopic images of undifferentiated and differentiated MSCs in transmitted light. **a** Undifferentiated MSCs, incubated in standard growth medium **b** MSCs on day 21 of osteogenic differentiation (*Alizarin Red S staining*) **c** MSCs on day 21 of chondrogenic differentiation (*Alcian blue staining*). The image size is 1289 × 964 μm (1392 × 1041 pixels)

MSC differentiation was identified by cellular morphology and specific staining. Before differentiation the MSCs had a spindle-shaped morphology and the cell population was homogeneous. By day 21 the differentiated cells had become polygonal in morphology. Alizarin Red S staining of the cells on day 21 of osteogenic differentiation showed dense extracellular matrix calcification. Alcian blue staining of the cells on day 21 of chondrogenic differentiation showed the presence of glycosaminoglycans (Fig. 1). The cells not incubated in osteogenic and chondrogenic media retained their spindle-shaped morphology through to day 21 and did not show any specific staining with the corresponding dyes.

**Fig. 2:**
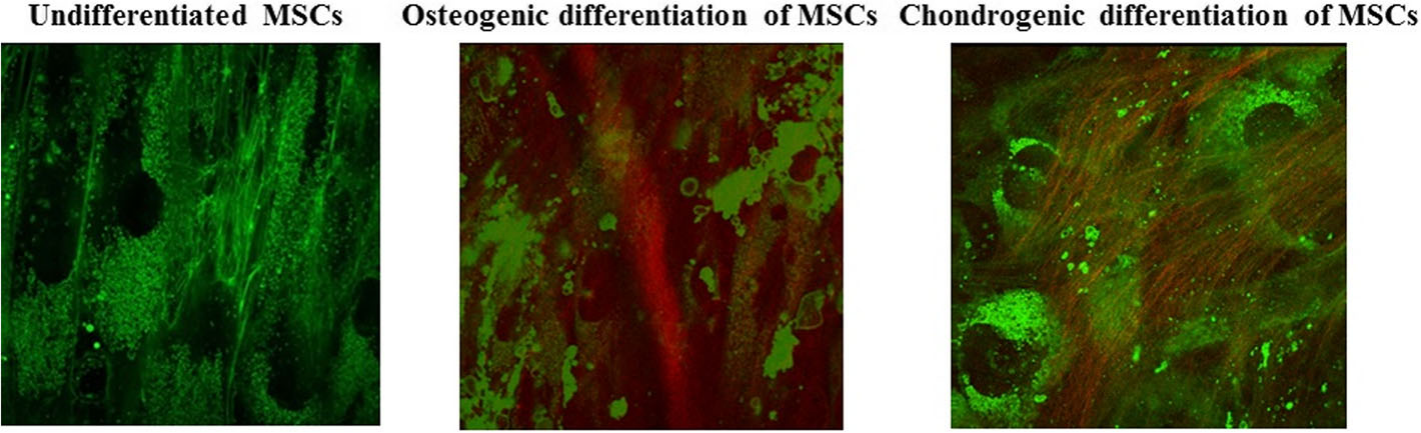
Overlapped two-photon excited autofluorescence (*green*) and SHG (*red*) images of undifferentiated MSCs, and MSCs during osteogenic and chondrogenic differentiation on day 21. The image size is 130 × 130 μm (512 × 512 pixels). *MSCs* mesenchymal stem cells

Using SHG microscopy we showed the presence of collagen fibers in chondrogenically differentiating cells at all time points of differentiation (7, 14, 21 days). In the case of osteogenic differentiation, collagen fibers were detected only on day 21 of differentiation. In undifferentiated cells collagen was not detected at all time points of examination (Fig. 2).

### Optical redox ratio during MSC differentiation

To estimate the rate of metabolic activity of the cells during osteogenic and chondrogenic differentiation, the fluorescence intensities of NAD(P)H and FAD were measured and represented as the redox ratio (FAD/NAD(P)H).

**Fig. 3:**
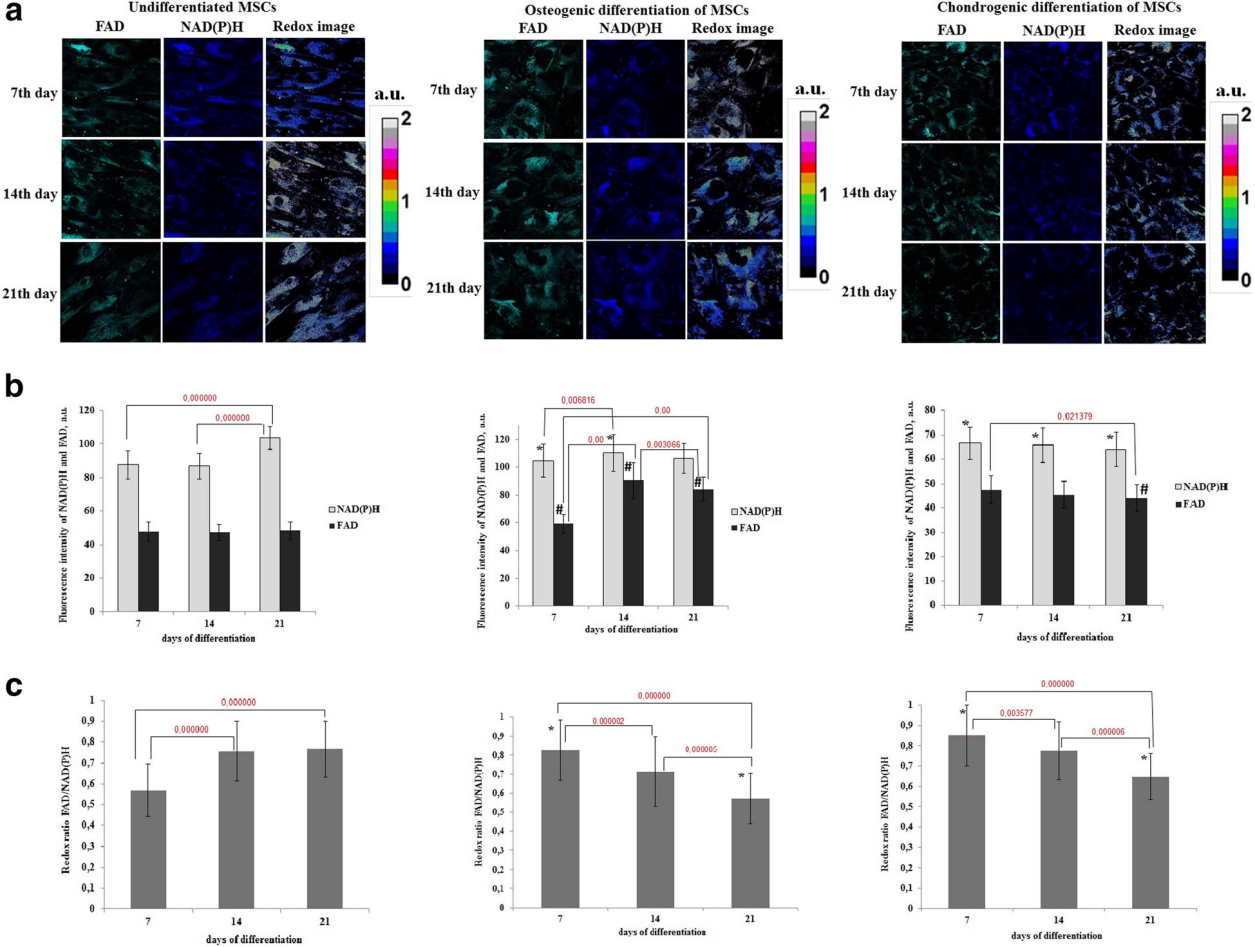
Redox ratio of FAD/NAD(P)H in differentiating MSCs. **a** Fluorescence and optical redox images of undifferentiated MSCs and MSCs during osteogenic and chondrogenic differentiation. Fluorescence of FAD is shown in *green*, fluorescence of NAD(P)H in *blue*. **b** Dynamics of the fluorescence intensity of NAD(P)H and FAD in undifferentiated MSCs and MSCs during osteogenic and chondrogenic differentiation, mean ± SD. ^*^Statistically significant difference on NAD(P)H with undifferentiated MSCs on the same day; ^#^statistically significant difference on FAD with undifferentiated MSCs on the same day **c** Dynamics of the ratio in undifferentiated MSCs and MSCs during osteogenic and chondrogenic differentiation, mean ± SD. ^*^Statistically significant difference with undifferentiated MSCs on the same day. For NAD(P)H: excitation - 750 nm, detection - 455–500 nm; for FAD: excitation - 900 nm, detection - 500–550 nm. The image size is 213 × 213 μm (1024 × 1024 pixels). *FAD* oxidized form of flavin adenine dinucleotide, *MSCs* mesenchymal stem cells, *NAD(P)H* reduced nicotinamide adenine dinucleotide (phosphate)

Representative dynamics of the ratios in differentiating MSCs are shown in Fig. 3a. The analysis of fluorescence intensities of NAD(P)H and FAD showed that during both differentiations FAD makes the main contribution to the redox ratio changes (Fig. 3b). NAD(P)H fluorescence intensity did not change in chondrogenic differentiation, and slightly increased on day 14 in osteogenic differentiation.

In the early stage of osteogenic and chondrogenic differentiation (day 7) a significant increase in the redox ratio was detected (0.83 ± 0.16 and 0.85 ± 0.15 vs 0.57 ± 0.12; *p* <0.000000). Subsequently the redox ratio gradually decreased in both cases, and by day 21 was lower than in the undifferentiated control cells (0.57 ± 0.13 and 0.65 ± 0.11 vs 0.77 ± 0.13; *p* <0.000000) (Fig. 3c). In both differentiations the redox ratios were significantly different between all days of differentiation within each group (*p* = 0.000002, *p* = 0.000005, *p* = 0.000000 for osteogenic, *p* = 0.003557, *p* = 0.000006, *p* = 0.000000 for chondrogenic differentiations). In the undifferentiated control statistically significant differences were detected between 7 and 14, 7 and 21 days.

### FLIM of NAD(P)H during differentiation

Since NADH is involved mostly in bioenergy release, we analyzed the fluorescence lifetimes and lifetime contributions of the free and protein-bound forms of NADH as indicators of the energy metabolism in the MSCs. It is known, that a bias toward oxidative metabolism results in a higher lifetime value and a higher contribution from protein-bound NADH [16–18].

As NAD(P)H fluorescence combines the contributions from free NADH, bound NADH and bound NADPH [19], and NADPH can be involved in oxidative stress, often associated with stem cell differentiation [20], as a preliminary, we tested the applicability of the three-exponential model for the fit of the fluorescence decay curves in order to estimate the contribution of NADPH to the overall fluorescence of NAD(P)H. This model was developed for separating NADH and NADPH by Blacker et al. [21]. However, the use of the three-exponential fit did not improve χ2 (0.8–1.2) when compared with the bi-exponential fit, and displayed an insignificant number of areas with a fluorescence lifetime t_3_ = 4.4 ns, corresponding to NADPH (approximately 1–3% of the total area of the images) at any differentiation stage. This allowed us to conclude, that the contribution of NADPH is negligible, and that bi-exponential fitting is rational for the analysis of the FLIM data.

**Fig. 4:**
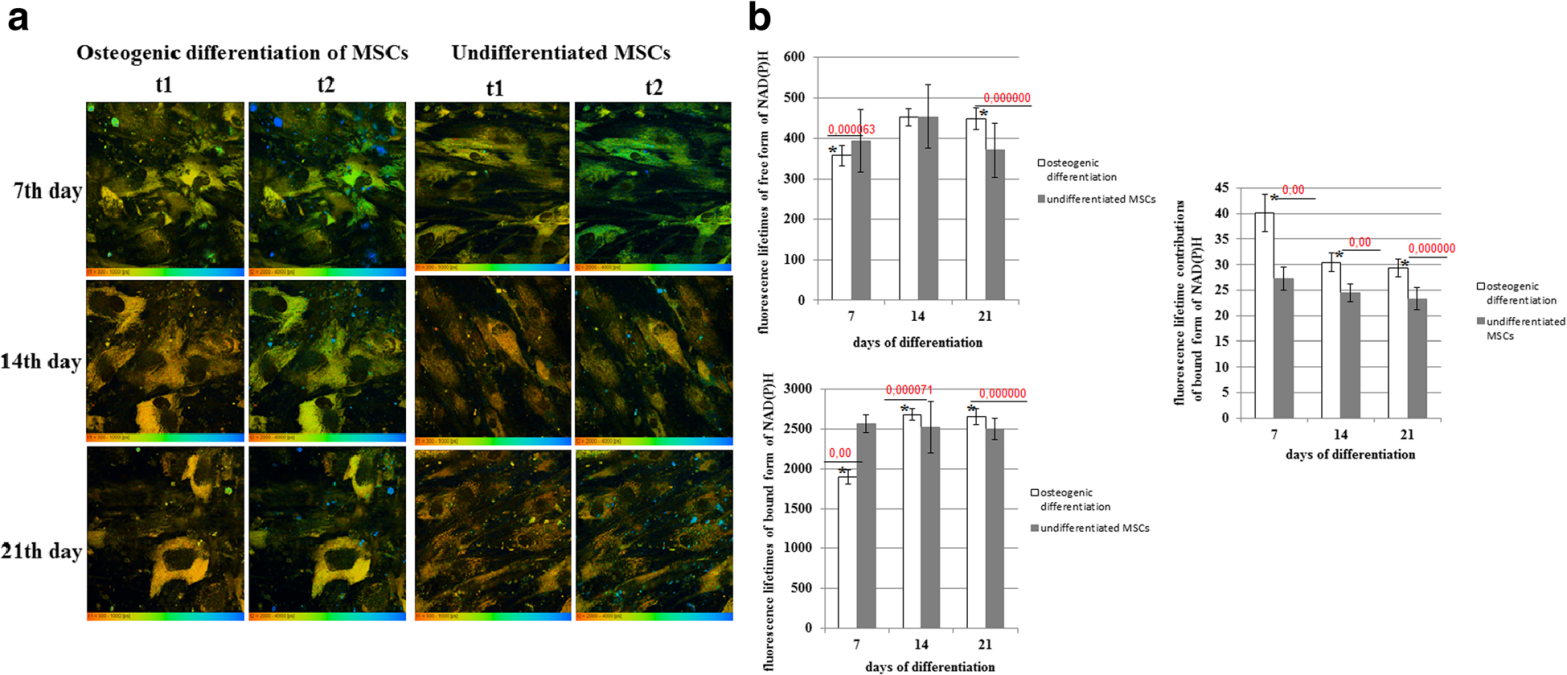
FLIM of NAD(P)H in MSCs during osteogenic differentiation. **a** Pseudocolor-coded images of the free (t1) and protein-bound (t2) forms of NAD(P)H. Field of view 213*213 μm (512*512 pixels). **b** Dynamics of the fluorescence lifetimes of free and protein-bound forms of NAD(P)H and fluorescence lifetime contributions of the protein-bound forms of NAD(P)H in MSCs. Mean ± SD. ^*^Statistically significant difference with undifferentiated MSCs on the same day. *P* values are shown. *MSCs* mesenchymal stem cells, *NAD(P)H* reduced nicotinamide adenine dinucleotide (phosphate)

It was found that, for osteogenic differentiation on day 7, the fluorescence lifetimes of the free (t1) and bound (t2) forms of NAD(P)H were lower than in the undifferentiated control. Further observation revealed a small, but statistically significant, increase of the short and long lifetimes of NAD(P)H (Fig. 4a, b) in differentiating cells.

The fluorescence lifetime contribution of protein-bound NAD(P)H (a2) was higher than in the undifferentiated MSCs at all time points during the differentiation, indicating, in general, a more oxidative state. The maximum elevation of bound NAD(P)H was detected in MSCs on 7 day of differentiation, which, along with an increased redox ratio at this time point, indicates a metabolic switch toward a more oxidative status compared with that of the undifferentiated cells. Subsequently, the contribution of protein-bound NAD(P)H in osteogenically differentiated MSCs gradually decreased (Fig. 4b), probably due to a bias toward more glycolytic metabolism.

**Fig. 5:**
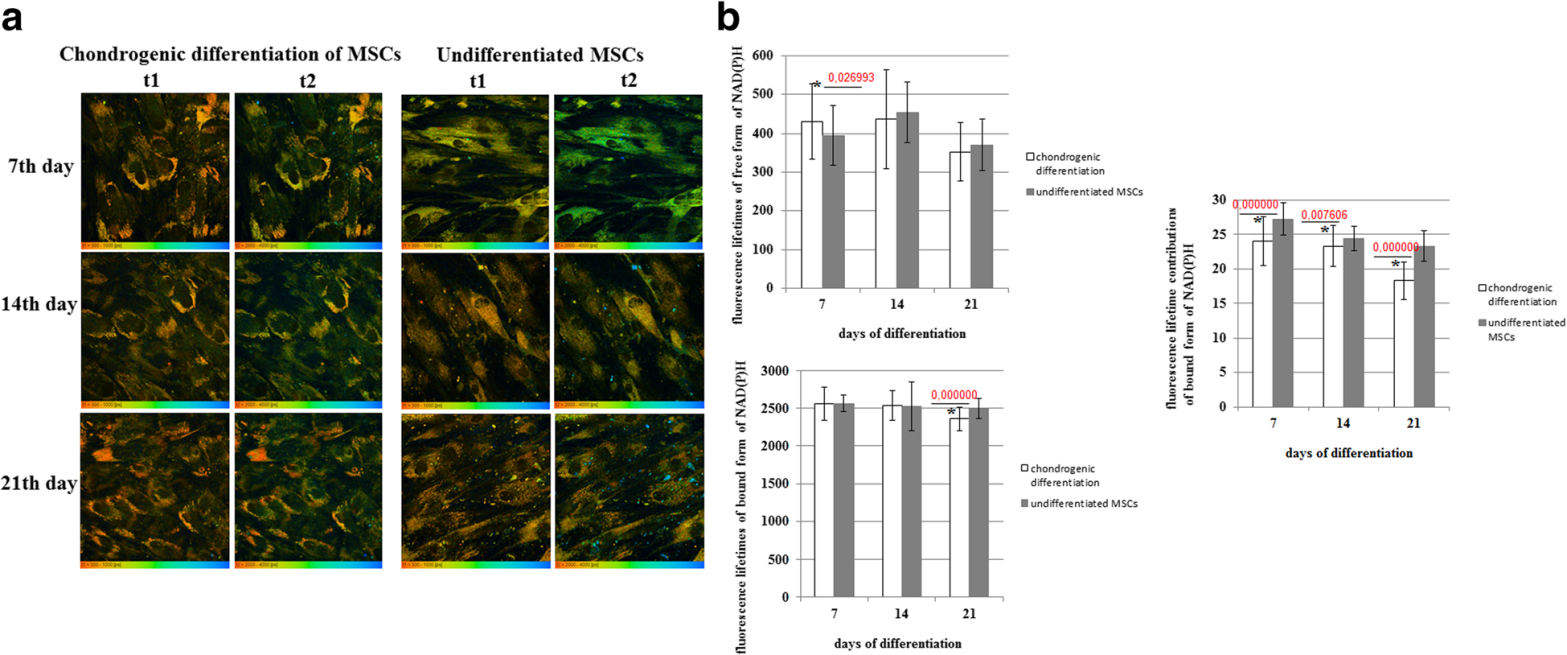
FLIM of NAD(P)H in MSCs during chondrogenic differentiation. **a** Pseudocolor-coded images of the free (t1) and protein-bound (t2) forms of NAD(P)H. Field of view 213*213 μm (512*512 pixels). **b** Dynamics of the fluorescence lifetimes of free and protein-bound forms of NAD(P)H and fluorescence lifetime contributions of the protein-bound forms of NAD(P)H in MSCs. Mean ± SD. ^*^Statistically significant difference with undifferentiated MSCs on the same day. *P* values are shown. *MSCs* mesenchymal stem cells, *NAD(P)H* reduced nicotinamide adenine dinucleotide (phosphate)

In the case of chondrogenic differentiation, the fluorescence lifetime of the free (t1) form of NAD(P)H only increased on day 7. The fluorescence lifetime of bound NAD(P)H (t2) did not change until day 21, when it slightly decreased (Fig. 5a, b).

The contribution of bound NAD(P)H (a2) in differentiating cells was less than in the non-differentiating ones throughout the observations, showing their more glycolytic status. In the process of differentiation the a2 value gradually decreased, with a statistically significant difference from the control at all differentiation stages, pointing to a shift toward glycolysis (Fig. 5b).

Therefore, along with decreasing redox ratios, these results suggest a shift to a more glycolytic metabolism in both types of differentiation.

### FLIM of FAD during differentiation

Although FAD plays an essential role in mitochondrial respiration, it is also involved in a number of other cellular pathways, including biosynthetic processes, and these are pronounced in differentiating stem cells. It was therefore hard to predict whether its behavior would correlate with the trajectories in energy metabolism or not.

During osteogenic differentiation the fluorescence lifetimes of free (t2) and protein-bound (t1) FAD were lower on day 7 than in the undifferentiated MSCs, but they then increased, exceeding the corresponding control values on days 14 and 21 of differentiation.

**Fig. 6:**
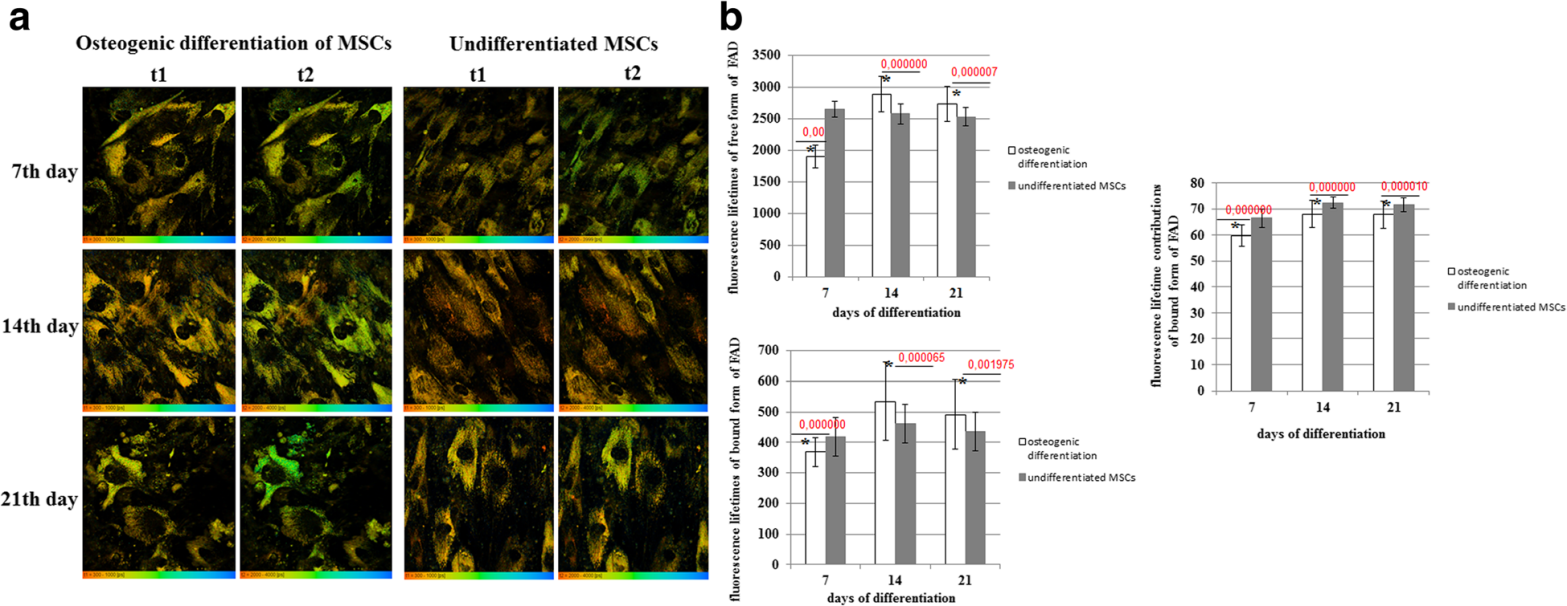
FLIM of FAD in MSCs during osteogenic differentiation. **a** Pseudocolor-coded images of the protein-bound (t1) and free (t2) forms of FAD. Field of view 213*213 μm (512*512 pixels). **b** Dynamics of the fluorescence lifetimes of free and protein-bound forms of FAD and fluorescence lifetime contributions of the protein-bound forms of FAD in MSCs. Mean ± SD. ^*^Statistically significant difference with undifferentiated MSCs on the same day. *P* values are shown. *FAD* oxidized form of flavin adenine dinucleotide, *MSCs* mesenchymal stem cells

The fluorescence lifetime contribution of bound FAD (a1) was lower at all time points compared with the undifferentiated cells. However, the a1 value increased (insignificantly) in osteogenically differentiated MSCs from day 7 to day 21 (Fig. 6a, b).

In chondrogenic differentiation the fluorescence lifetimes of FAD did not change appreciably. The only small difference (decrease) from the undifferentiated cells was detected on day 21.

**Fig. 7:**
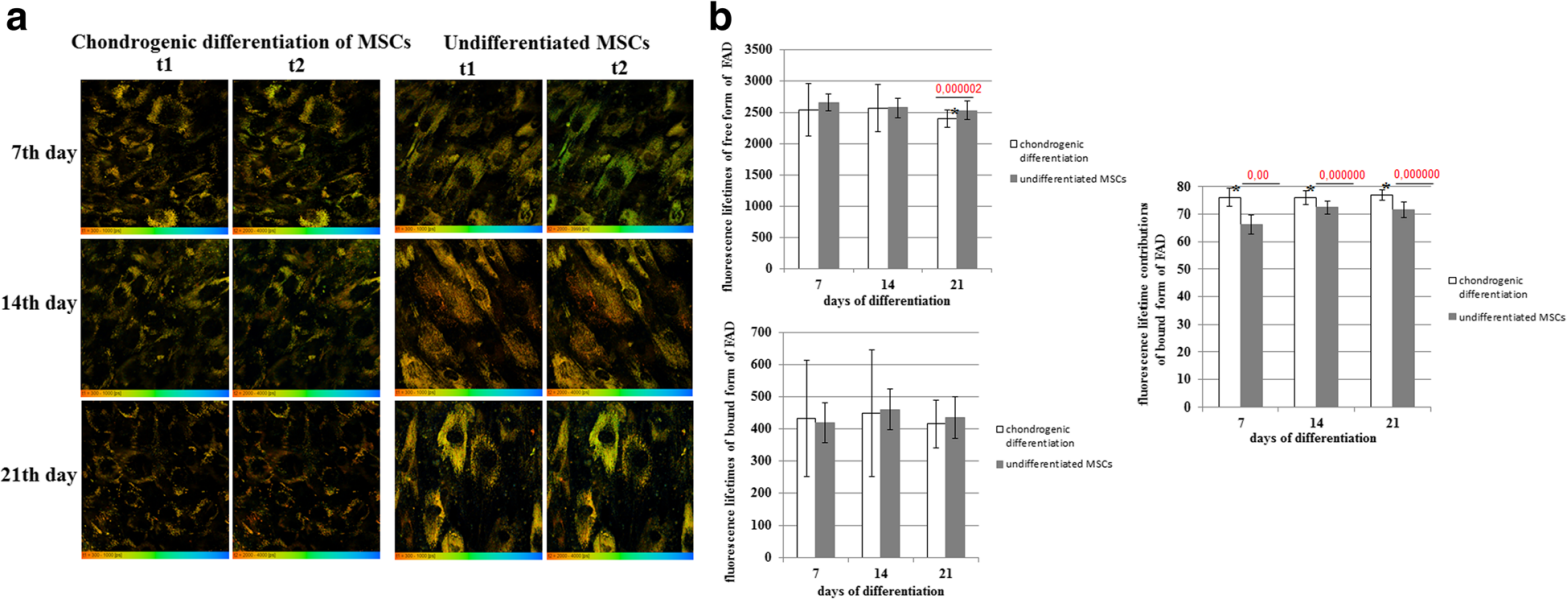
FLIM of FAD in MSCs during chondrogenic differentiation. **a** Pseudocolor-coded images of the protein-bound (t1) and free (t2) forms of FAD. Field of view 213*213 μm (512*512 pixels). **b** Dynamics of the fluorescence lifetimes of free and protein-bound forms of FAD and fluorescence lifetime contributions of the protein-bound forms of FAD in MSCs. mean ± SD. ^*^Statistically significant difference with undifferentiated MSCs on the same day. *P* values are shown. *FAD* oxidized form of flavin adenine dinucleotide, *MSCs* mesenchymal stem cells

A statistically significant increase in the protein-bound FAD contribution (a1) (in comparison with the undifferentiated MSCs, which showed no changes from day 7 to day 21) was revealed during chondrogenic differentiation (Fig. 7a, b).

Therefore, the parameters of the FAD fluorescence lifetimes did not agree with our observations related to energy metabolism. In the case of osteogenic differentiation with a marked oxidative state of the MSCs at all stages, the bound FAD fluorescence lifetime and contribution were reduced. On the contrary, during chondrogenic differentiation in MSCs having a more glycolytic state, the bound FAD fluorescence lifetime and contribution increased. Taken together, these data suggest that a separate FAD-involving pathway is activated.

## Discussion

The FLIM technique implemented on the TCSPC principle is a powerful tool for stem cell biology. In this study we investigated the metabolic changes in living MSCs during osteogenic and chondrogenic differentiation, using two-photon fluorescence microscopy and FLIM. Based on the data for the FAD/NAD(P)H redox ratio and on the fluorescence lifetimes of protein-bound NAD(P)H, we registered a metabolic shift toward a more glycolytic status in the process of MSC differentiation. The difference was that, in osteogenic differentiation, an increase in oxidative phosphorylation preceded the shift to the glycolytic status in the process of such MSC differentiation. The fluorescence lifetime characteristics of FAD indicated the stimulation of an unknown metabolic pathway, where protein-bound FAD participates.

An increasing body of evidence has appeared that oxidative metabolism and the upregulation of mitochondrial function are essential for the successful differentiation of MSCs [22, 23].

We detected an increased redox ratio of FAD/NAD(P)H and an increased contribution of protein-bound NADH at the beginning of osteogenic differentiation (day 7) that verifies an elevation of the OxPhos rate. Active OxPhos is likely required to meet the high ATP demands needed for the extensive biosynthesis of extracellular matrix protein during osteogenesis. Chen et al. reported the activation of OxPhos in MSCs during osteogenic differentiation [24]. In a recent study, Guntur et al. performed bioenergetic profiling during osteogenic differentiation of calvarial osteoblasts, and also observed activation of OxPhos [25]. In other studies, where no changes in the OxPhos rates were detected in osteogenically differentiating MSCs [14, 26], this may be associated with the use of culture media containing pyruvate, as this can feed directly into the mitochondrial citric acid cycle. However, Shum et al. showed that while OxPhos is activated during MSC osteogenesis, glycolytic activity does not decrease [13]. The authors reported that OxPhos becomes active only at the stage of osteogenic differentiation when proliferation is less pronounced, and this is in agreement with our data. Moreover Long’s group suggested a continuous dependence on glycolysis during osteogenic differentiation [26].

In chondrogenic differentiation we observed a decreasing FAD/NAD(P)H redox ratio and a decreasing contribution of protein-bound NADH, which enabled us to suggest that differentiated cells (chondrocytes) rely predominantly on glycolysis, although the use of oxidative phosphorylation cannot be excluded. Indeed, the production of lactate normally seen under normoxia, referred to as the Warburg effect, has previously been demonstrated for chondrocytes that reside under hypoxia in vivo [27, 28]. Wang et al. also reported higher rates of glycolysis following chondrogenic differentiation when compared to proliferative MSCs [29]. In the study by Pattappa et al. co-determination of azide-sensitive oxygen consumption and glycolysis indicated that chondrogenic cultures utilize oxidative phosphorylation for, at most, 13% of their total ATP production on day 21, thereby resulting in a predominantly glycolytic metabolism (89%) [14]. However, the authors associated the suppressed oxidative phosphorylation with the culture conditions rather than with the chondrogenic state per se.

The underlying mechanism of a metabolic shift to glycolytic status during osteogenic and chondrogenic differentiation may be related to enhanced collagen synthesis that requires a more reduced state of the cells. For example, increased lactate production has been associated with collagen deposition by fibroblasts during wound healing [30] and by MSCs during osteoblastic differentiation. In the study by Ghukasyan et al. [31] collagen deposition was detected on day 21 after the addition of osteoblastic induction factors. However, the organization of collagen into larger bundles was significantly greater only by day 28. In our study using SHG microscopy we showed the presence of collagen fibers in chondrogenically differentiating cells at all time points of differentiation (7, 14, 21 days). In case of osteogenic differentiation, collagen fibers were detected only on day 21 of differentiation.

While energy metabolism involving NAD(P)H is quite well described for stem cells using the FLIM method [16, 32, 33], the dynamics of FAD lifetimes during stem cell differentiation have been very poorly investigated. The problem with FAD, in this context, is that it is involved in many other metabolic pathways besides energy metabolism, which complicates interpretation of the results. Previously, we had detected a decreased contribution of protein-bound FAD during adipogenic differentiation of MSCs against the background of a shift toward more oxidative metabolism [18, 34], which is likely associated with the beta-oxidation of fatty acids, where FAD is reduced to FADH_2_. If no other pathways contribute much to the state of the FAD, the decreased lifetime contribution of bound FAD is associated with a greater emphasis on glycolytic metabolism, as was shown by Skala et al. for epithelial precancer [35].

In our study we detected an elevated contribution of protein-bound FAD when, presumably, the glycolysis rate was increased (chondrogenic differentiation) and a reduced contribution where there was initially more oxidative metabolism, and a subsequent increase during a shift toward a greater emphasis on glycolysis (osteogenic differentiation). We speculate that the inconsistency of the parameters of energy metabolism and FAD may be connected with the involvement of FAD in the alpha-ketoglutarate dehydrogenase complex. One of the reactions of the TCA cycle occurring outside the mitochondria is the cleavage of alpha-ketoglutarate to succinate and carbon dioxide. This reaction is involved in the hydroxylation of the prolyl and lysyl residues of protocollagen, a step in the synthesis of collagen. It is possible that increase of the fluorescence intensity of FAD during MSCs differentiation was due to participation of FAD in this biosynthetic pathway.

However, the dynamics of the free and bound forms of FAD during stem cell differentiation require further appropriate metabolic measurements.

Currently, multiphoton microscopy is applied for in vivo tracking of stem cells labelled with fluorescent protein [36]. Label-free two-photon FLIM of metabolic cofactors is a promising strategy for analysis of stem cell behavior in living animals. Although FLIM technique has been successfully used on stem cell cultures [37], three-dimensional models of spheroids [38] and scaffolds [39], and on tissue slices [40], its capabilities have been poorly explored in stem cell research in vivo so far. The possibility of using FLIM for analysis of the relationship between metabolic oscillations and circadian phase within epidermal stem cells in live mice at the single-cell level has been demonstrated recently in the study by Stringari et al. [41]. The results of our study provide a basis for label-free monitoring of stem cell differentiation in vivo with the use of two-photon FLIM of metabolic status.

## Conclusions

In this study, probing of the metabolic status of MSCs during osteogenic and chondrogenic differentiation was implemented for the first time with the use of optical metabolic imaging of the two cofactors - NAD(P)H and FAD. Our data suggest that biosynthetic processes, associated, presumably, with the synthesis of collagen, drive energy metabolism in differentiating cells, and promote a metabolic shift from a more oxidative to a more glycolytic state. Understanding of the metabolic peculiarities of stem cells and the changes accompanying the differentiation process is extremely important for the development of new treatment strategies in regenerative medicine and stem cell therapy.

## Abbreviations

ATP: Adenosine triphosphate
CoA: Coenzyme A
CoQ: Coenzyme Q
DNA: Deoxyribonucleic acid
FAD: Oxidized form of flavin adenine dinucleotide
FADH: Reduced flavin adenine dinucleotide
FBS: Fetal bovine serum
FLIM: Fluorescence lifetime imaging microscopy
LipDH: Lipoamide dehydrogenase
MSCs: Mesenchymal stem cells
NAD(P)H: Reduced nicotinamide adenine dinucleotide (phosphate)
NAD+: Oxidized form of nicotinamide adenine dinucleotide
OxPhos: Oxidative phosphorylation
PDHC: Pyruvate dehydrogenase complex
SHG: Second harmonic generation
TCA cycle: Tricarboxylic acid cycle
TCSPC: Time-correlated single photon counting
